# Evaluation of pain and opioid consumption in local preemptive anesthesia and the erector spine plane block in thoracoscopic surgery: A randomized clinical trial

**DOI:** 10.1590/0100-6991e-20223291-en

**Published:** 2022-08-25

**Authors:** IGHOR PALLU, SOFIA DE SOUZA BOSCOLI, TANIA ZALESKI, DIANCARLOS PEREIRA DE ANDRADE, GUILHERME RODRIGO LOBO CHERUBINI, ALEXANDRA INGRID DOS SANTOS CZEPULA, JULIANO MENDES DE SOUZA

**Affiliations:** 1 - Faculdades Pequeno Principe, Curso de Medicina - Curitiba - PR - Brasil; 2 - Hospital Nossa Senhora das Graças, Departamento de Anestesiologia - Curitiba - PR - Brasil; 3 - Hospital Nossa Senhora das Graças, Departamento de Cirurgia Torácica - Curitiba - PR - Brasil

**Keywords:** Thoracic Surgery, Video-Assisted, Pain, Postoperative, Abuse, Opioid, Anesthesia, Local, Anesthesia, Cirurgia Torácica Vídeoassistida, Dor Pós-Operatória, Anestesia Local, Anestesia, Analgésicos Opioides

## Abstract

**Objective:**

assess pain and opioid consumption in patients undergoing anesthetic techniques of spinal erector plane block and local anesthetic block in video-assisted thoracic surgery in the immediate postoperative period.

**Methods:**

ninety-two patients undergoing video assisted thoracic surgery were randomized to receive ESPB or BAL before starting the surgical procedure. Using the numerical verbal scale, the primary outcome assessed was the patient’s pain in the immediate postoperative period (POI). The secondary outcome comprises the assessment of opioid consumption in the IPP by quantifying the medication used in an equianalgesic dose of morphine expressed in milligrams, in the immediate post-anesthetic recovery period, 6h, 12h, and 24h after surgery.

**Results:**

the EVN scores in the LBA and ESPB group in the POI had a mean of 0,8 (±1,89) vs 0,58 (±2,02) in the post-anesthesia care room (REPAI), 1,06 (±2,00) vs 1,30 (±2,30) in 6 hours of POI, 0,84 (±1,74) vs 1,19 (±2,01) within 12 hours of POI and 0,95 (±1,88) vs 1 ( ±1,66) within 24 hours of POI, all with p>0.05. Mean opioid consumption in the BAL and ESPB groups in the POI was 12.9 (± 10.4) mg vs 14.9 (±10.2) mg, respectively, with p = 0.416. Sixteen participants in the ESPB group and seventeen in the BAL group did not use opioids during the first 24 hours of the PO analyzed.

**Conclusion:**

local anesthesic block and ESP block techniques showed similar results in terms of low pain scores and opioid consumption during the period evaluated.

## RESUMO

**Objetivo:**

avaliar a dor e o consumo de opioides dos pacientes submetidos a técnicas anestésicas de bloqueio do plano eretor da espinha (ESPB) e bloqueio anestésico local (LBA) em cirurgia torácica vídeo assistida no período pós-operatório imediato (POI).

**Métodos:**

noventa e dois pacientes submetidos a cirurgia torácica videotoracoscópica foram randomizados aleatoriamente para receberem ESPB ou LBA antes do início do procedimento cirúrgico. O desfecho primário avaliado foi a dor do paciente no POI através da escala verbal numérica. O desfecho secundário avaliou o consumo de opioides através da quantificação da medicação usada em dose equianalgésica de morfina expressa em miligramas, no período de recuperação pós-anestésica imediata, 6h, 12h e 24h após a cirurgia.

**Resultados:**

os escores da Escala Verbal Numérica de dor (EVN) no grupo LBA e ESPB no POI, respectivamente, tiveram média de 0,8 (±1,89) vs 0,58 (±2,02) na sala de recuperação pós anestesia (REPAI), 1,06 (±2,00) vs 1,30 (±2,30) em 6 horas do POI, 0,84 (±1,74) vs 1,19 (±2,01) em 12 horas do POI e 0,95 (±1,88) vs 1 ( ±1,66) em 24 horas do POI, todos com p>0,05. O consumo médio de opioides no grupo LBA e ESPB foi de 12,9 (±10,4) mg vs 14,9 (±10.2) mg, respectivamente, com p=0.416. Dezesseis participantes do grupo ESPB e dezessete do grupo LBA não utilizaram opioides durante as primeiras 24 horas do PO.

**Conclusões:**

as técnicas de bloqueio LBA e ESPB apresentaram resultados semelhantes em termos de baixos escores de dor e consumo de opioides durante o período avaliado.

## INTRODUCTION

Effective pain management in the postoperative period has a great impact on the recovery of patients undergoing Video Assisted Thoracic Surgery (VATS), allowing for a reduction in hospital stay, lower rates of consumption of opioid analgesics, and optimized patient recovery^1^ ^-^ ^3^. Pain in the immediate postoperative period (IPO) after VATS directly impacts the patient’s ventilatory capacity and mobility, increasing the rates of complications such as atelectasis, pulmonary infection, and venous thromboembolic disease^1^ ^-^ ^3^. Furthermore, it increases the immediate risks of developing hypoxemia, hypercapnia, increased cardiac work, and arrhythmias^4^.

A multimodal anesthetic approach, combining a parenteral analgesic method with regional or local anesthetic blocks, such as preemptive anesthesia, has been effective in controlling pain in the IPO, leading to a reduction in opioid consumption in this period and consequently minimizing the side effects of these medications^4^. This concept of analgesia refers to the administration of anesthetics before the surgical incision or manipulation of the site, to reduce sensitization and hyperalgesia at the level of the central nervous system^1^ ^,^ ^4^.

Preemptive anesthesia in VATS can be performed in several ways, including the local injection modality, applied to the incision sites and insertion of drains and trocars, or other areas prone to painful sensation due to surgical manipulation^5^. Another possible modality is the Erector Spinae Plane Block (ESPB), which according to recent research, promotes unilateral analgesia similar to that of an epidural block, without causing blockage of the autonomic nervous system (ANS)^6^.

Among the anesthetic methods available, the epidural block is the most used, however, due to side effects, risks, and, sometimes, the impossibility of performing the technique, other methods have been tested. Therefore, due to the importance of promoting efficient and safe analgesia to the patient, in addition to the concern regarding the use of opioids after surgical procedures, we conducted this comparative study between ESPB anesthetic block and local anesthetic block (LAB), to evaluate pain and opioid consumption in the IPO of patients undergoing these techniques in VATS procedures.

## METHODS

This is a randomized, blind clinical trial, carried out at the Hospital Nossa Senhora das Graças (HNSG) in Curitiba, Paraná State, Brazil, approved by the Ethics in Research Committee of the Faculdades Pequeno Príncipe (FPP), under opinion number 4,425,817. This study was previously registered in the Brazilian Clinical Trials Registry system under the reference code U1111-1264-5523.

We included patients classified by the American Society of Anesthesiologist (ASA) between 1 and 3, aged 18 years or older, with surgical indication for the treatment of any thoracic diseases, who could be performed by minimally invasive, uni or multiportal techniques, carried out from December 2020 to November 2021.

We excluded patients with absolute contraindication to VATS procedures, anesthetic drugs used during the procedure, and analgesic medications used in the postoperative period. We also excluded patients with a history of illicit drug or opioid abuse, with a medical diagnosis of dementia, delirium, or other conditions that affect verbal response, pregnant women, emergency procedures, and patients with difficulty understanding the pain scales used in the protocol.

The randomization process was performed using a table of random numbers, where patients who received an odd number were allocated to the intervention group (ESPB), and those who received an even number were allocated to the control group (LAB). Each participant received individualized surgical treatment, using an already established surgical technique, considered safe and effective, according to a specific indication for each case, regardless of the group in which they were allocated.

### Anesthetic induction, sedation, and perioperative management

After initial pre-oxygenation, all patients underwent total intravenous anesthesia, with continuous infusion of remifentanil 0.2-05mcg/kg/min and target-controlled infusion (TCI) propofol at a dose of 2.5-3.5ng/ml, to maintain a bispectral index (BIS) between 40-60. For neuromuscular blockade, cisatracurium was used at induction at a dose of 0.15mg/kg, with further doses of 0.05mg/kg to maintain a sequence of four stimuli (TOF) of 0. All patients in the study were intubated with a double-lumen cuffed endobronchial tube, between 35-39F, maintaining single-lung ventilation during the procedure. The anesthetic blocks used in the study were performed only after general anesthesia, as is already routine in the hospital service, thus maintaining the blinding of the group allocation to patients^2^ ^-^ ^7^. 

### LAB Technique (Control Group)

Patients randomized to the control group received a solution with 15mg of 0.5mg/ml levobupivacaine (corresponding to 30ml), added to 4mg of 4mg/ml dexamethasone disodium phosphate (corresponding to 1ml) and 150µg of 150µg/ml clonidine hydrochloride (corresponding to to 1ml), which were injected into the surgical incisions using 20 or 22-gauge needles, always by the same team of two surgeons. Complete local block was performed by applying the solution to the skin, costal periosteum, and parietal pleura, approximately five minutes before the surgical incisions to position the trocars^1^ ^-^ ^8^.

### ESPB Technique (Intervention Group)

Anesthetic block in patients randomized to the intervention group occurred with the patient in a sitting or lateral decubitus position, after general anesthesia, with a high-frequency linear transducer placed in cephalocaudal or longitudinal orientation over the paramedian line, to visualize the ribs and pleura at the level of the of 4^th^ thoracic vertebra (T4). After, the transducer was slid medially to visualize the T4 transverse process (rectangular hyperechoic line, with posterior acoustic shadow), with a deepening of the pleura in the image.

**Figure 1:**
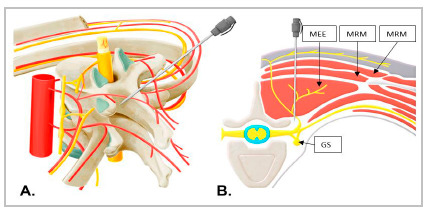
A. Conceptual illustration of the location of anesthetic application for erector spinae plane block (ESPB). B. The anesthetic solution is injected into the fascial plane between the erector spinae muscle, near the lateral end of the transverse process. Application in this site allows the anesthetic to spread anteriorly into the paravertebral space through the intertransverse connective tissue and reach the epidural space through the intervertebral foramen. There is also craniocaudal and lateral spread of the solution through the fascia below the erector spinae muscle. ESM= erector spinae muscle; RMM= rhomboid major muscle; TM= trapezius muscle; SG= sympathetic ganglion. (Source: Adapted by the authors, from Chin & El-Boghdadly, 2021).

At this point, it is possible to identify the trapezius, rhomboid major, and erector spinal muscles. Using a Stimuplex A100 B.Braun^®^ needle on the skin, with an angle of 30° to 45° about the ultrasound beam, facing the transverse process (Figure 1A), the needle was inserted up to the interfascial plane to the erector spinae muscles group, where, under continuous ultrasound guidance, a solution of 20ml of 0.25% levobupivacaine without vasoconstrictor was injected (Figure 1B)^2^ ^,^ ^7^ ^,^ ^9^. 

### Postoperative

To control moderate and severe pain, was prescribed 0.05mg/kg of ideal weight of morphine or another opioid in an equianalgesic dose (tramadol or nalbuphine), administered when requested by the patient, to maintain the verbal score of the pain scale in values less than four^10^.

The primary outcome of this study was the patient’s pain level in the immediate postoperative period and throughout the first 24 hours after the procedure, at specific times, using a verbal numerical scale (VNS). Patients were to measure the pain at four different times, still in the Anesthetic Recovery Unit (REPAI) or at the time of the patient’s arrival at the ICU, at 6, 12, and 24 hours after the procedure. The VNS quantifies the pain on a scale from zero to ten, zero corresponding to absence of pain, and ten, the most intense pain possible^7^.

The secondary outcome was the cumulative consumption of opioids during the first 24 hours of the PO period, expressed as an equianalgesic dose of IV morphine in milligrams (MEQs). Patients who were discharged before 24 hours of PO had MEQs dose evaluated until discharge.

Data such as sex, age, initial diagnosis, surgery performed, surgery time in minutes, and PO drain use were also documented and used for comparative measures.

### Statistical analysis

The sample size calculation was performed using the Welch-Satterthwaite T-Testt^12^.

According to the study by Zheng et al., the mean pain scores in the postoperative period of patients undergoing VATS procedures was 5.4 points on a scale ranging from 0 to 10^13^. Given that a reduction of at least 1.5 points on this scale can be considered as clinically significant, we can assume that the mean of the first group is 5.4 points, and that of the second one, 3.9 points, with a standard deviation of 2.4^13^. Thus, one can calculate the sample size needed to evaluate each group as being 42 patients, with a sample power of 80% and a significance level of 5% (α=0.05).

For quantitative variables, the distribution of normality was verified using the Shapiro-Wilk test and the results were reported using the mean (± standard deviation) or median (interquartile range). As for qualitative variables, the values of each group were expressed as an absolute number (% percentage of the total)^14^.

To verify the statistical significance between the data, were applied the Mann-Whitney, t, ANOVA, Kruskal-Wallis, or Friedman tests. For all tests, we considered values of p<0.05 sufficient to reject the null hypothesis and deem the result statistically significant^17^.

All statistical analyses and construction of graphs and tables were performed using the JAMOVI^®^ statistical software, version 2.2.1^15^.

## RESULTS

**Figure 2:**
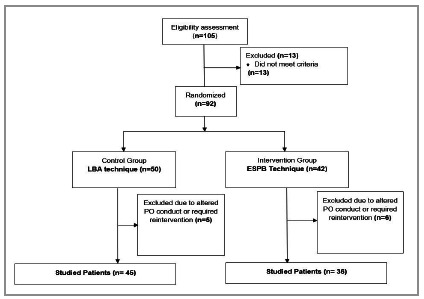
CONSORT diagram of study patient flow.

The flowchart of the Consolidated Standards of Reporting Trails (CONSORT)16 of this study is represented in Figure 2. At the end of the study, 81 participants were followed up and had their results analyzed.

Categorical data such as age, sex, duration of surgery, and type of procedure performed were compared between groups, as shown in Table 1. In none of the groups there was any documented adverse reaction or complication related to the drugs or anesthetic block techniques.

**Table 1:** Comparison of patient characteristics and VATS surgical procedures performed in the LAB and ESPB groups.

Variable	LBA (n=45)	ESPB (n=36)
Age (years)	58.5 (± 17.4)	57.8 (± 17.7)
Women	24 (53.3%)	19 (52.8%)
Surgery Time (minutes)	132.5 (±8.4)	133.9 (±88.5)
Drain usage	31 (68.9%)	22 (61.1%)
Surgery side (left)	24 (53.3%)	19 (52.8%)
Type of Surgical Procedure (VATS)		
Bullectomy	2 (4.4%)	1 (2.8%)
Pulmonary Decortication	1 (2.2%)	1 (2.8%)
Pulmonary Lobectomy	7 (15.6%)	10 (27.8%)
Pleuroscopy	13 (28.9%)	2 (5.6%)
Pneumonectomy	0 (0%)	1 (2.8%)
Mediastinal Tumor Resection	3 (6.7%)	13 (36.1%)
Wall Tumor Resection	1 (2.2%)	0 (0%)
Pleural Tumor Resection	0 (0%)	1 (2.8%)
Pulmonary Segmentectomy	14 (31.1%)	7 (19.4%)
Sympathectomy - Reconstruction	4 (8.9%)	0 (0%)
Pulmonary Segmentectomy	14 (31.1%)	7 (19.4%)
Sympathectomy - Reconstruction	4 (8.9%)	0 (0%)

Data described as mean (± standard deviation) or n (%). Source: the authors (2021).

**Figure 3:**
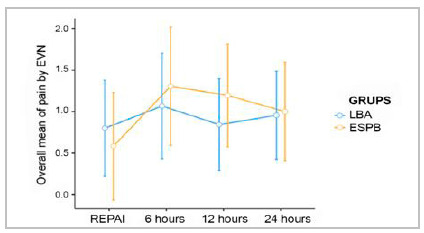
Graph of Friedman’s test (unpaired ANOVA) comparing pain between LAB and ESPB groups over the IPO time. Values represented as mean and 95% confidence interval. Source: The authors (2021).

The VNS pain scores in both groups throughout the analyzed time interval showed no statistically significant differences, with p values >0.05 at all times, as seen in Table 2 and Figure 3 of the Friedman test plot, where the absolute value of the pain score reported by the patients was considered. Approximately 75.3% of patients in both groups had no pain or mild pain (1-4 VNS) at the time of assessment.

**Table 2:** Primary and Secondary Outcomes.

Variable	LBA	ESPB	p-value
Pain (≥1 VNS) at some point in the PO	29 (64.4%)	29 (80.6%)	0.110**
Pain in REPAI/ICU (VNS)	0.8 (±1.89)	0.58 (±2.02)	0.089*
Absence of pain (0)	34 (75.6%)	33 (91.7%)	
Mild Pain (1-4)	8 (17.8%)	0	
Moderate Pain (5-7)	2 (4.4%)	2 (5.6%)	0.069**
Severe Pain (8-10)	1 (2.2%)	1 (2.8%)	
Pain 6h PO (NVS)	1.06 (±2.00)	1.30 (±2.30)	0.646*
Absence of pain (0)	31 (68.9%)	23 (63.9%)	
Mild Pain (1-4)	10 (22.2%)	9 (25%)	0.971**
Moderate Pain (5-7)	3 (6.7%)	3 (8.7%)	
Severe Pain (8 to 10)	1 (2.2%)	1 (2.8%)	
Pain 12h PO (NVS)	0.84 (±1.74)	1.19 (±2.01)	0.260*
Absence of pain (0)	33 (73.3%)	22 (61.1%)	
Mild Pain (1-4)	7 (15.6%)	12 (33.3%)	0.146**
Moderate Pain (5-7)	5 (11.1%)	2 (5.6%)	
Severe Pain (8-10)	0 (0%)	0 (0%)	0.766*
Pain 24h PO (NVS)	0.95 (±1.88)	1 (±1.66)	
Absence of pain (0)	30 (66.7%)	23 (63.9%)	0.081**
Mild Pain (1-4)	13 (28.9%)	9 (25%)	
Moderate Pain (5-7)	0 (0%)	4 (11.1%)	
Severe Pain (8-10)	2 (4.4%)	0 (0%)	
Number of patients who used opioids in the PO	28 (62.2%)	20 (55.6%)	0.544**
Total postoperative opioid consumption (mg) in 24h	12.9 (±10.4)	14.9 (±10.2)	0.416*

Data are described as mean (± standard deviation) or n (%);*Mann-Whitney; **Chi-square. Source: The authors (2021).

Sixteen participants in the ESPB group and seventeen in the LAB group did not use opioids during the first 24 hours of the PO analyzed. There was no significant difference in 24-hour opioid consumption between the two groups, with p=0.416.

When analyzing only the 21 patients who underwent lobectomies or segmentectomies in the LAB group, the mean pain in the REPAI/ICU was 0.62 (±1.80), and after 24 hours of IPO, 1.14 (±1.93). For the ESPB group, of the 17 patients who underwent the same procedures, the mean pain in the REPAI/ICU was 0.94 (±2.68), and 24 hours after the procedure, 0.94 (±1.34). The mean opioid consumption in these patients was 8.19mg (±8.44) and 7.06mg (±9.49) for the LAB and ESPB groups, respectively. When comparing these variables between groups, there was no statistically significant difference in any of them, with p values >0.05.

## DISCUSSION

ESPB has the ability to block the dorsal and ventral branches of the spinal nerves through the craniocaudal spread of up to four dermatomes above and below the injection site, increasing its analgesic efficiency during surgery and in the postoperative period^17^ ^,^ ^18^. On the other hand, LAB, used in several minimally invasive surgical procedures such as gynecological laparoscopy, cholecystectomy, and arthroscopies, is still poorly described and applied in VATS procedures^8^. In the rare studies found, the results of this technique have proved to be attractive, since its performance is simple and quick, and can be used in a wide range of procedures, from chest drainage to lobectomies^1^ ^,^ ^8^ ^,^ ^19^ ^,^ ^20^.

We identified no similar study in the literature, in the form of a randomized clinical trial comparing LAB and ESPB in patients undergoing VATS, evaluating pain and opioid consumption by patients in the IPO.

In this study, both groups showed similar results in pain control. In both the ESPB and LBA groups, the results pointed to adequate analgesic efficacy after VATS. The VNS pain score recorded by 75.3% patients in both groups was null or did not exceed score 4 at any of the postoperative follow-up moments, corroborating the findings of other researchers^8^ ^,^ ^21^ ^,^ ^22^. 

When analyzing the groups separately according to the data in Table 2, the average pain in the REPAI/ICU of the ESPB group was lower than that described in the literature, whose values for this moment were 1.90 (±1, 34)^23^ and 1.50 (±0.80)^22^, whilst in our series the reported value was 0.58 (±2.02). At the 6^th^ hour of the IPO, the mean pain in VNS found by other authors was 3.33 (±0.48)^23^, higher than our finding, where the mean was 1.30 (±2.30). Within 24 hours of the IPO, we found a mean of 1.00 (±1.66), an intermediate value to those described in the literature, with means of 0.27 (±0.52)^23^ and 2.5 (±0.7)^24^.

Of the few studies available that assess the pain of patients in the postoperative period of VATS procedures when submitted to local anesthetic block, most of them analyze only patients who underwent segmentectomies or lobectomies. Based on this principle, from our patients in the LAB group who underwent only these two procedures, the mean pain in the REPAI and in 24 hours of the IPO were 0.62 (±1.80) and 1.14 (±1.93), respectively. When compared to other studies, the average found for REPAI was 8.3 (±2.1) and 2.3 (±1.3) in 24 hours of the IPO5. In addition, regardless of the surgical procedure performed with this type of anesthetic block, we observed no significant difference in postoperative pain control, with the overall mean of pain on the VNS in the REPAI/ICU of 0.8 (±1.89) and 0.95 (±1.88) 24 hours after surgery.

Opioid consumption can also be an objective variable for pain assessment, since pain quantification in scales can suffer social, psychological, and cultural interferences^25^. When evaluating opioid consumption in this study, we identified no significant differences between the groups. However, when comparing the results of this research with those of other authors, the mean consumption of opioids in the IPO was 29.3mg in patients undergoing ESPB in a prospective study using the same drug and concentration we used in the blocking protocol, but in this study the mean consumption of opioids was approximately 50% lower (14.9 ±10.2mg)^2^. Another study, using the same technique, described the consumption of 29.39 mg (±3.8) in the first 24 hours^26^. 

As for the LAB group, in a clinical study evaluating patients undergoing only lobectomies and segmentectomies who received 0.5% ropivacaine local anesthetic blockade, the mean drug consumption in 24 hours was 42mg (± 29.58)^19^. Of the twenty-one patients in this research who received preemptive local anesthesia and underwent these procedures, the mean opioid consumption was 8.19mg (± 8.44).

In none of the studies discussed above the non-use of opioids was described by some portion of patients. In our research, seventeen patients from the LBA group and sixteen from the ESPB group did not use this class of drugs during the 24 hours analyzed. During this period, the excessive use of the substance is responsible for important side effects that have a negative impact on the patient’s recovery, such as respiratory depression, increased risk of bleeding, and gastrointestinal disorders^27^. Moreover, there is great concern about the risk of dependence on this type of substance arising from inadequate pain management in the IPO, with a 44% increased risk for long-term use of opioids^28^.

It is important to emphasize that there are limitations in this clinical trial. Despite randomization, there was a difference in the number of patients undergoing different types of surgeries between the study and control groups. It is known that procedures such as lobectomies may be associated with more significant postoperative pain and may influence results^2^ ^,^ ^27^. In addition to being a subjective variable, pain can be influenced by numerous factors, altering individual perception, making it necessary to have a greater number of participants in each group in order to render results more accurate. Another limitation was the assessment of the patients’ pain only at four postoperative moments and the non-assessment of the patient’s pain on chest movement tests (such as forced coughing). Also, we did not evaluate pain and opioid consumption after the first 24 hours of PO.

## CONCLUSIONS

There was no statistically significant difference between the groups undergoing LAB and ESPB blocks in terms of postoperative pain control and opioid consumption in the IPO of VATS procedures. Both preemptive anesthesia techniques were effective in pain control and capable of promoting low intravenous use of opioids. Eventual differences between the techniques need to be studied with a greater number of patients and new pain assessment protocols.
